# Flavonoid Profiles of Two New Approved Romanian *Ocimum* Hybrids

**DOI:** 10.3390/molecules25194573

**Published:** 2020-10-07

**Authors:** Fanica Balanescu, Maria Daniela Ionica Mihaila, Geta Cârâc, Bianca Furdui, Costel Vînătoru, Sorin Marius Avramescu, Elena Lacramioara Lisa, Mihaela Cudalbeanu, Rodica Mihaela Dinica

**Affiliations:** 1 Faculty of Sciences and Environment, Department of Chemistry Physical and Environment, “Dunarea de Jos” University of Galati, 111 Domneasca Street, 800201 Galati, Romania; fanica.balanescu@ugal.ro (F.B.); ionicamariadaniela@gmail.com (M.D.I.M.); geta.carac@ugal.ro (G.C.); 2 Faculty of Medicine and Pharmacy, “Dunarea de Jos” University of Galati, 35 Al. I. Cuza Street, 800010 Galati, Romania; elena.lisa@ugal.ro; 3 Vegetable Research and Development Station Buzău, 23 Mesteacănului Street, 120024 Buzau, Romania; costel_vinatoru@yahoo.com; 4 Faculty of Chemistry, Department of Organic Chemistry, Biochemistry and Catalysis, University of Bucharest, 90–92 Soseaua Panduri, 050663 Bucharest, Romania; sorin_avramescu@yahoo.com; 5 University of Agronomic Science and Veterinary Medicine, 59 Marasti Blvd, 011464 Bucharest, Romania; 6 National Institute for Research and Development in Environmental Protection–INCDPM, 294 Splaiul Independentei, 060031 Bucharest, Romania

**Keywords:** flavonoids, HPLC-DAD quantification, antioxidant activity, cyclic voltammetry, Romanian *Ocimum* hybrids

## Abstract

Basil (*Ocimum* spp.) is a traditional herbal medicine abundant in antioxidants such as phenolic compounds. As part of a diet, this herb is proved to have some roles in decreasing the risk of cancer, and in the treatment of inflammation and neurodegenerative diseases. This study aims to explore the total phenolic and flavonoid content of two new basil hybrids growing in Romania, namely “Aromat de Buzau” (AB) and “Macedon” (MB). The antioxidant capacity of those two species was also analyzed by DPPH and cyclic voltammetry. Six different flavonoids, such as catechin (+), rutin, hyperoside, naringin, naringenin, and genistein, were separated, identified, and quantified by HPLC–DAD chromatography, for the first time, from romanian basil hybrids. The main flavonoid of the extracts was found to be naringin which is present in the highest amount (26.18 mg/kg) in “Aromat de Buzau” (*O. basilicum*) methanolic extract. These results suggest that dietary intake of these new hybrids can be a source of antioxidant compounds.

## 1. Introduction

To prevent the radical chain of oxidation reactions, antioxidants are often added to foods, acting by inhibiting the beginning and multiplication steps, leading to the close of the reaction and the delay of the oxidation process. Finding natural antioxidants to replace synthetic compounds because of safety concerns and the increase of consumer’s preferences for natural antioxidants is one of the main concerns of the food industry [1,2].

There is strong evidence that oxidative stress is caused by an imbalance between prooxidants and antioxidants. The reactive species, such as reactive oxygen species (ROS), nitrogen species (RNS), or electrophilic species (RES), and the cells antioxidative defense systems, play a central role in the pathogenesis of several diseases, including neurodegenerative diseases, kidney diseases, and diabetes, cancers, or cardiovascular diseases. To counteract this stress, the nuclear factor erythroid 2-related factor 2 (Nrf2) plays a major role in reducing oxidative damage through a series of small antioxidant molecules and maintaining intracellular redox homeostasis [3,4,5,6,7].

The most important natural antioxidants with important biological activities are polyphenols, compounds that have attracted the interest of researchers for the prevention and treatment of various diseases [8,9]. Flavonoids are a family of polyphenolic plant metabolites [10] having various functions in plants, acting as antimicrobial agents, as plant protection photoreceptors, and UV protectors, but they are also responsible for a range of pharmacological properties, including antioxidant, antiallergic, anti-inflammatory, antimicrobial, anti-viral, antithrombotic, or antioncogenic activities [11,12,13]. The biological activity of flavonoids depends on their structural class, the degree of hydroxylation, substitutions and conjugations, and on the degree of polymerization [13,14].

Many flavonoidic compounds, such as rutin, naringin, naringenin, or genistein, have been reported to have physiologic benefits, including inhibition of tumor angiogenesis, protection from cardiovascular disease, promotion of neural protection, anti-angiogenic activities in inflammatory, chronic, and metabolic diseases [15,16], and nitric oxide scavenging activity [10]. Many of these benefits coming from their special antioxidant and free radical inhibition properties [17].

The *Ocimum* genus belongs to the *Lamiaceae* family that contains over 150 cultivated aromatic perennial herb species, spread all over tropical and temperate regions, used in diet and to treat different types of diseases [2,18,19,20]. *Ocimum basilicum* (*O. basilicum*) is popularly known as sweet basil and has been reported in many fields, e.g., agriculture, food, ornamental, religious, and pharmacology, due to its volatile aromatic compounds [19,20,21]. *Ocimum citriodorum* (*O. citriodorum*), a natural hybrid between sweet basil (*O. basilicum*) and African basil (*O. americanum*), is known as lemon basil due to the specific strong aroma of lemon and is often used in the Mediterranean and Asian cuisines because of its odor and flavor, and also used as a raw material for the chemical, pharmaceutical, and food industries [20,22].

Basil was brought to Europe long time ago by Alexander the Great [20]. Since 1996, various hybrids of basil were acclimatized in Buzau, Romania [20]. Due to their beneficial healthy effects, the consumption of culinary herbs has increased worldwide, so the knowledge of new polyphenol-rich herbs is of increasing interest. We report here, for the first time, the evaluation of alcoholic extracts of two basil hybrids from Romanian cultivars: “Aromat de Buzau” (*O. basilicum*) and “Macedon” (*O. citriodorum*) from the point of view of polyphenol and flavonoid compounds. Therefore, we explored the antioxidant potential of Romanian basil hybrids by developing methods for HPLC-DAD flavonoid quantifications, and for antioxidant capacity evaluation through free radical inhibitory activity and cyclic voltammetry.

## 2. Results

### 2.1. Phytochemical Characterization of the Romanian Varieties of Basil

**Figure 1:**
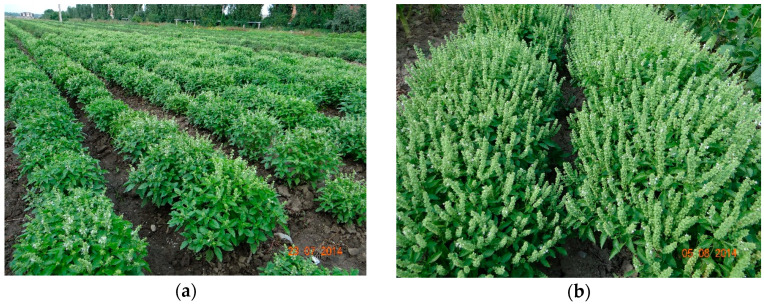
*O. basilicum* hybrids: “Aromat de Buzau” (**a**) “Macedon” (**b**).

The goal of the present research was the quantification of biological compounds such as phenolic acid and flavonoid and the evaluation of the antioxidant effects of two Romanian basil hybrids extracts. The diversity and the content of phenolic acids and flavonoids were determined for methanolic and ethanolic extracts of two Romania *Ocimum* hybrids, “Aromat de Buzau” and ”Macedon”, respectively (Figure 1), by developing also an HPLC-DAD method.

#### 2.1.1. Polyphenolic Acid Content

**Figure 2:**
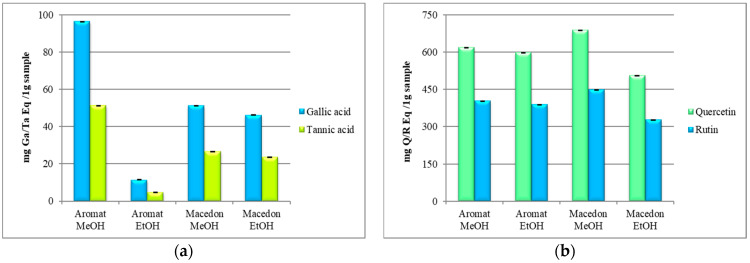
The total content of the phenolic acids (**a**) and flavonoids (**b**) extracted from “Aromat de Buzau” (*O. basilicum*) and “Macedon” (*O. citriodorum*) extracts. The results were expressed in equivalents of gallic acid and tannic acid per 1 g sample, and quercetin and rutin per 1 g sample, respectively.

The total phenolic acids based on the Folin–Ciocalteu micromethod were determined for ethanolic and methanolic extracts of *Ocimum* hybrids [23], and this led to the results which are presented in Figure 2a.

#### 2.1.2. Flavonoid Content

The same solvents, methanol and ethanol, were also used for the evaluation of the extraction efficiency of flavonoids, expressed as quercetin (Q) and rutin (R). The results of the four basil extracts showed a higher amount of the total flavonoid contents than phenolic acids contents. Some variations were observed according to the plant species and the extraction solvents used (Figure 2b).

### 2.2. Antioxidant Potentials of the Romanian Varieties of Basil

To explore the relation between the composition of extracts of antioxidant compounds and their antioxidant capacities, two different techniques (DPPH assay and cyclic voltammetry) were used.

#### 2.2.1. DPPH Assay

Polyphenol compounds of the plant origin have been shown to exert antioxidant effects [24]. Any compound, in low concentrations compared with those of an oxidizable substrate which presents significantly delays or prevents oxidation of the substrate is an antioxidant [25]. DPPH is a violet stable free radical which interacts with antioxidant compounds, and through an electrons-proton transfer process it is inactivated and changes its color from violet to yellow [24]. By increasing the concentration of phenolic compounds and the degree of hydroxylation of the phenolic compounds, their DPPH radical scavenging activity also increases and can be defined as antioxidant activity [24].

**Figure 3:**
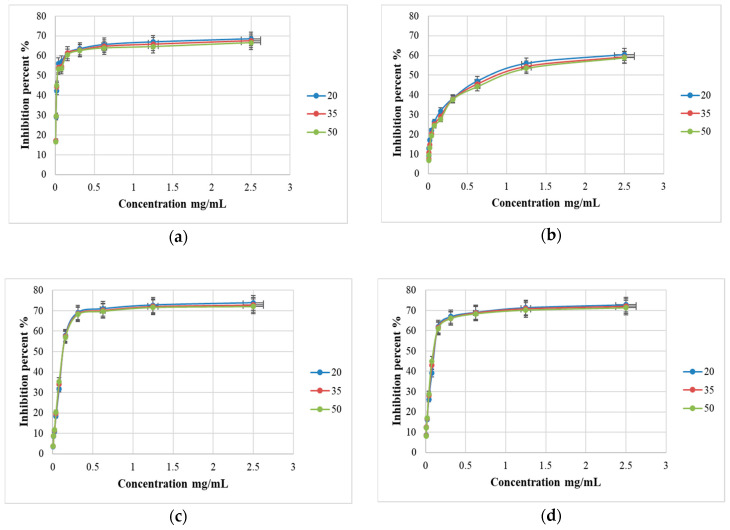
The DPPH inhibition percent of the (**a**) *O. basilicum* (”Aromat de Buzau”) methanolic extract, (**b**) *O. basilicum* (“Aromat de Buzau”) ethanolic extract, (**c**) *O. citriodorum* (“Macedon”) methanolic extract, and (**d**) *O. citriodorum* (“Macedon”) ethanolic extract after 20 (blue), 35 (red) and 50 (green) minutes of incubation.

**Figure 4:**
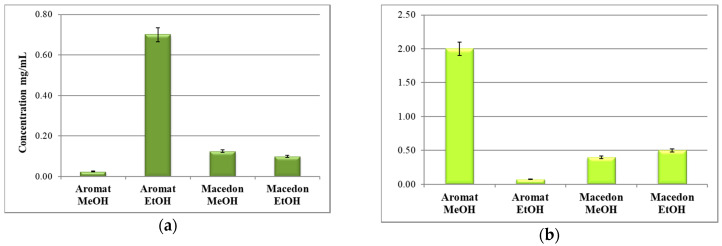
The IC_50_ values (**a**) and antioxidant capacity index (**b**) of the basil extracts.

Different concentrations of basil methanolic and ethanolic extracts, between 0.0048 and 2.5 mg/mL were used for the antioxidant DPPH assays and their IC_50_ values are presented (Figure 3 and Figure 4).

#### 2.2.2. Electrochemical Evaluation of Antioxidant Capacity by Cyclic Voltammetry

**Figure 5:**
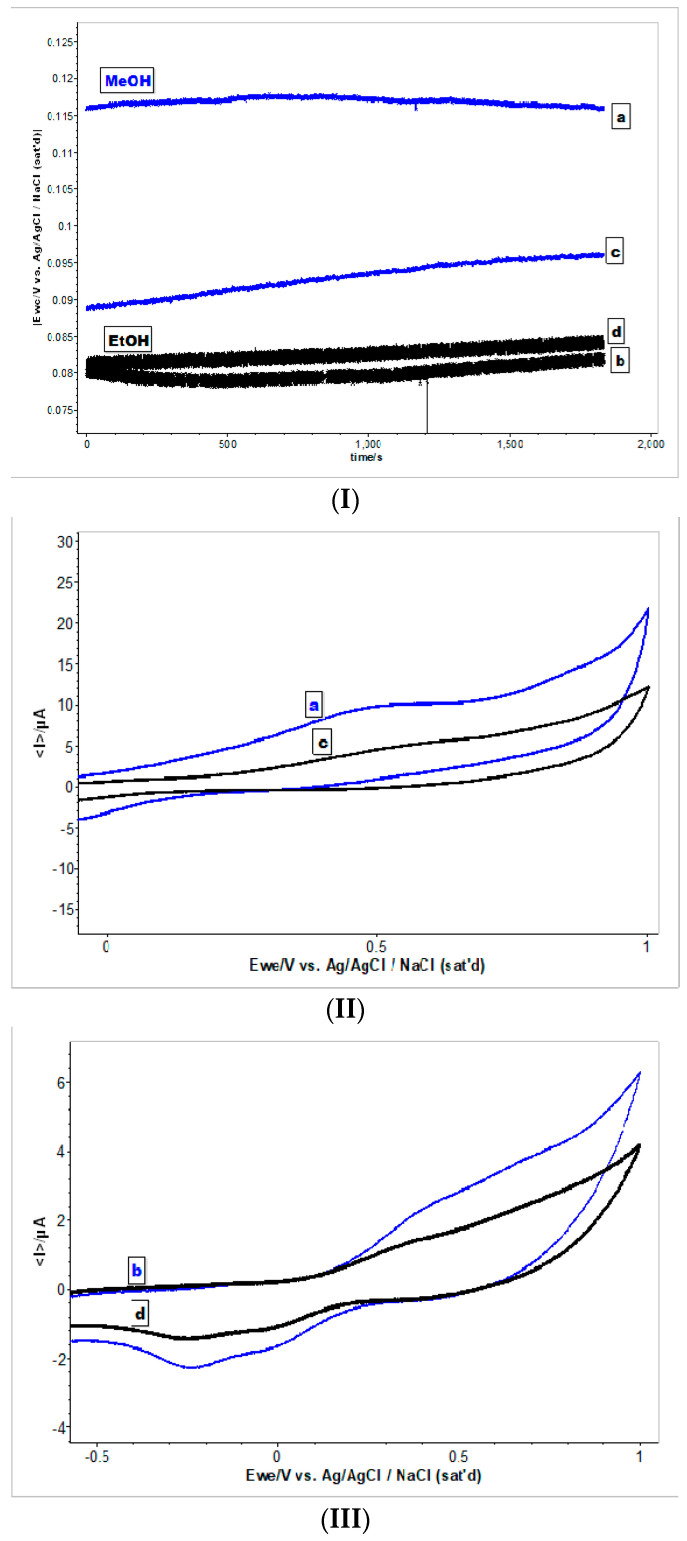
**(I)** OCV registered for basil methanolic extracts of AB (**a**) and MB (**c**) and, respectively for ethanolic extracts of AB (**b**) and MB (**d**). (**II**) Cyclic voltammograms recorded for basil methanolic extracts of AB (a-blue) and MB (c-black), E = ±1 V/Ag/Ag Cl_sat_, at 100 mVs^−1^. (**III**) Cyclic voltammograms recorded for basil ethanolic extracts of AB (c-blue) and MB (d-black), E = ±1 V/Ag/Ag Clsat, at 100 mVs^−1^.

The recorded OCV (open circuit voltage) of the extracts from both basil hybrids show a stable potential at 30 min with a slow difference in the values of potential between used solvents (Figure 5I). A more positive potential (0.115 V/Ag/AgCl_sat_) was determined for the AB (“Aromat de Buzau”) methanolic extracts, than for the MB (“Macedon”) methanolic extracts (0.095 V/Ag/AgCl_sat_). The potential of the ethanolic extracts was around of 80 mV/Ag/AgCl_sat_ and there were no significant differences between the basil samples.

Figure 5II,III shows the cyclic voltammograms of all basil samples and it is obvious that the voltammogram profiles of two hybrid species are different. In the *O. basilicum* (“Aromat de Buzau”) methanolic extract, around 0.5 V/Ag/AgClsat are biocompounds with potential intensity activity of I_a_ 60 μA, with circa 50% more active than in the *O. citriodorum* (“Macedon”) methanolic extract. In the ethanolic extracts, even the intensity activity is less than in methanolic AB (“Aromat de Buzau”) extract compared with the MB (“Macedon”) extract showing a larger band for the electronic changes starting from 0.25 V/Ag/AgCl_sat_ till 0.80 V/Ag/AgCl_sat_ (Figure 5III).

**Figure 6:**
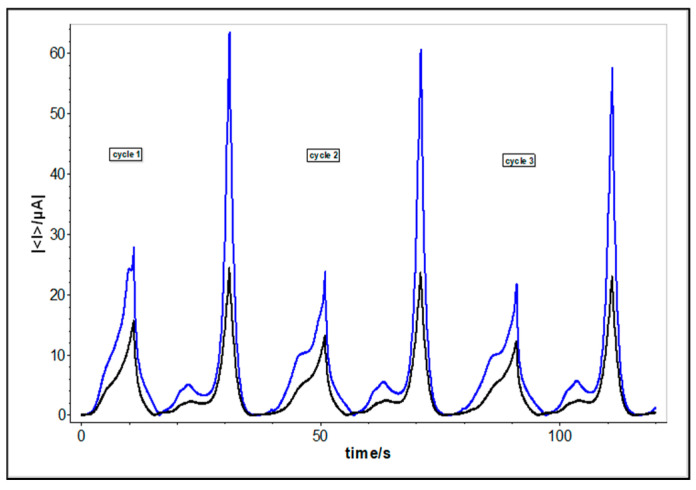
Evolution of current vs time in cyclic voltammograms recorded for basil methanolic extracts of AB (blue) and MB (black), E = ±1 V/Ag/Ag Cl_sat_, at 100 mVs^−1^; 3 cycles.

The CV’s recorded for basil hybrid extracts after 24 h indicated an increase in the Ia current values. For the both methanol extracts, the current also indicates a decreased use of values when the experiment cycle is repeated, which suggests that, the active compounds are still in the samples (Figure 6) [26].

**Figure 7:**
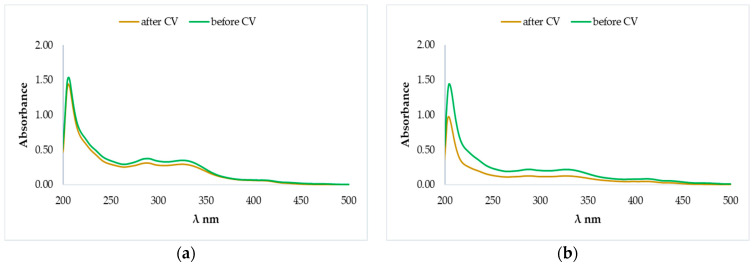

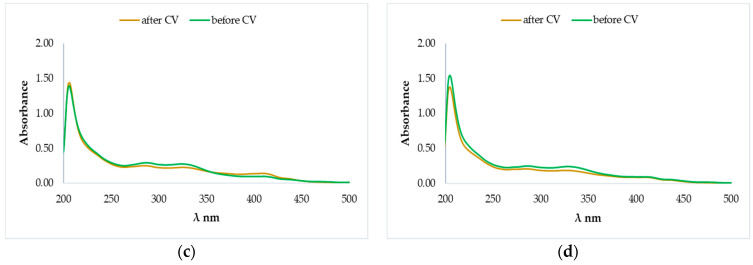
UV-VIS spectra recorded for the samples before and after cyclic voltammetry experiments: “Aromat de Buzau” methanolic extract (**a**) and ethanolic extract (**b**), “Macedon” methanolic extract (**c**) and ethanolic extract (**d**).

Figure 7 shows that the UV bands of basil extracts before and after CV analysis overlap. The characteristic wavelengths of each UV-Vis spectrum proved the presence of flavonoidic compounds in the analyzed extracts. The presence of naringin in the Romanian basil hybrid extracts was confirmed by HPLC-DAD quantification using naringin as standard and it was observed in both Romanian basil varieties, with a λ_max_ at 228 nm and other characteristic signals in the range of 250~320 nm [27,28].

### 2.3. HPLC-DAD Separation, Identification and Quantification of Flavonoid Compounds

To analyze the major contributors on the antioxidant activity of our plant matrices, HPLC-DAD analysis was performed. Several studies of *Ocimum* species have described the presence of bioactive compounds in different plant parts extracts [29]. In this research, the analysis of the secondary metabolites from flavonoid class was made quantitatively using an original the HPLC-DAD method. Generally, flavonoids have many roles and functions, their protective effects including anti-inflammatory, antioxidant, antiviral, and anti-carcinogenic properties [30,31].

**Figure 8:**
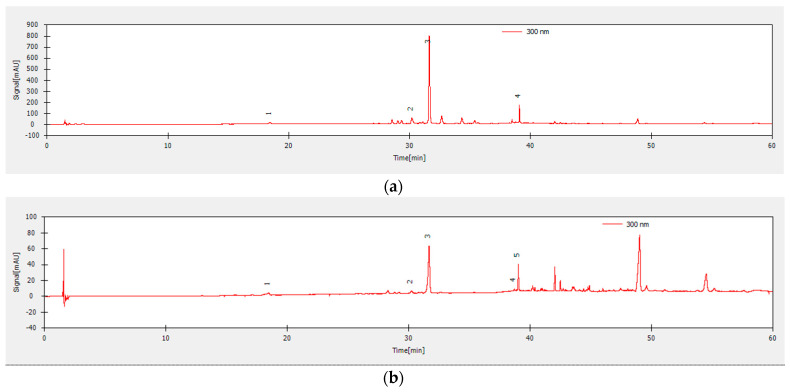

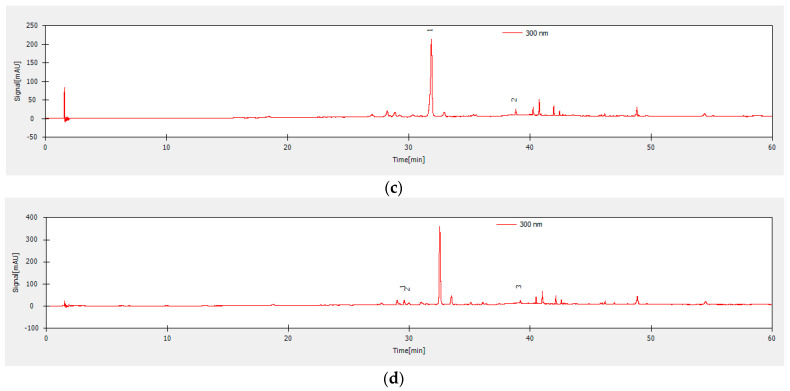
HPLC–DAD chromatograms of the samples: (**a**) “Aromat de Buzau” -methanolic extract (1—catechin, 2—rutin, 3—naringin, 4—genistein), (**b**) “Aromat de Buzau” ethanolic extract (1—catechin, 2—rutin, 3—naringin, 4—naringenin, 5—genistein), (**c**) “Macedon” methanolic extract (1—naringin, 2—naringenin), and (**d**) “Macedon” ethanolic extract (1—rutin, 2—hyperoside, 3—genistein) with detection at 300 nm.

The chromatographic separation, using reference compounds, revealed the presence in the Romanian basil extracts of six flavonoids, namely catechin (+), rutin, hyperoside, naringin, naringenin, and genistein (Figure 8). Other peaks could not be identified due to a lack of standards. The flavonoid quantification of the four methanolic and ethanolic basil extracts of the two different Romanian *Ocimum* hybrids are summarized in Table 1. The results showed that the naringin is the principal compound of basil extracts ranged from 9.30 ± 0.15 to 26.18 ± 0.23 mg/kg dry plant. The best quantities of naringin were recorded for both methanolic extract of basil hybrids, the concentration being three times higher in *O. basilicum* variety “Aromat de Buzau”.

## 3. Disscusion

The analyzed extracts of Romanian *Ocimum* hybrids were prepared by ultrasound solvent extraction which favors cell wall disruption and greater solvent penetration into the cells, accelerating the extraction.

### 3.1. Phytochemical Characterization of the Romanian Varieties of Basil

The extraction efficiency of polyphenols was evaluated for two different solvents, ethanol and methanol, based on total phenolic acids content expressed as gallic acid (GA) and tannic acid (Ta). The results revealed that, from the four basil extracts, the methanolic extract of “Aromat de Buzau” contain the highest quantity of phenolic compounds (96.32 ± 0.23 mg GaEq/1g sample and 51.16 ± 0.19 mg TaEq/1g sample, respectively) while the ethanolic extract of same hybrid contain the lower quantity of phenolic compounds (11.47 ± 0.12 mg GaEq/1g sample and 4.78 ± 0.05 mg TaEq/1g sample, respectively) (Figure 2a). By comparison, the phenolic acids present in the “Macedon” extracts showed a lower content than “Aromat de Buzau” methanolic extract, but higher contents than “Aromat de Buzau” ethanolic extract (51.24 ± 0.42 mg GaEq/1g sample and 26.51 ± 0.10 mg TaEq/1g sample, respectively for “Macedon” methanolic extract and 46.13 ± 0.31 mg GaEq/1g sample and 23.72 ± 0.12 mg TaEq/1g sample, respectively for “Macedon” ethanolic extract).

The results of the four basil extracts showed a higher amount of the total flavonoid contents than phenolic acids contents. Some variations were observed according to the plant species and the extraction solvents used (Figure 1b). “Aromat de Buzau” (*O. basilicum*) methanolic extract presents a flavonoid amount of 619.34 ± 4.98 mg QEq/1g sample and 402.43 ± 3.06 mg REq/1g sample, while the ethanolic extract contains 598.78 ± 2.63 mg QEq/1g sample and 388.91 ± 2.98 mg REq/1g sample, respectively. The total flavonoid amount of “Macedon” (*O. citriodorum*) methanolic extract was found to be 689.05 ± 5.78 mg QEq/1g sample and 448.18 ± 3.05 mg REq/1g sample while the contents of flavonoids for “Macedon” (*O. citriodorum*) ethanolic extract expressed as quercetin was 505.20 ± 3.34 mg QEq/1g sample and expressed as rutin was 327.50 ± 2.87 mg REq/1g sample.

### 3.2. Antioxidant Potentials of the Romanian Varieties of Basil

The results obtained by analyzing the Romanian basil hybrid samples showed that the DPPH radical scavenger activity is greatly influenced by the solvent extraction and the phytochemical composition (Figure 2). The antioxidant capacity of basil species is a useful parameter which correlates with the phytochemical determinations. The *O. basilicum* (“Aromat de Buzau”) methanolic extract showed free radical scavenging activity with an inhibition percent of 68 ± 0.98% while the ethanolic extract had a lower inhibition percent of 60 ± 0.85%. Also, the *O. citriodorum* (“Macedon”) methanolic extract showed a free radical scavenging activity with an inhibition percent of 73 ± 0.88% while the ethanolic extract had an inhibition percent of 72 ± 0.73%.

The DPPH scavenging IC_50_ values estimated for the Romanian basil hybrid extracts were of 0.025 ± 0.011 mg/mL (“Aromat de Buzau” methanolic extract), 0.700 ± 0.302 mg/mL (“Aromat de Buzau” ethanolic extract), 0.125 ± 0.121 mg/mL (“Macedon” methanolic extract), and respectively of 0.100 ± 0.097 mg/mL (“Macedon” ethanolic extract). Interestingly, a lower IC_50_ value shows a higher antioxidant activity in the basil extracts [26]. The highest antioxidant activity was registered for the *O. basilicum* spp., “Aromat de Buzau” methanolic extract, a result which can be correlated with its high phenolic acid contents (Figure 4a). Our results are in accordance with those from the literature data which showed strong scavenging activity of DPPH radical for the basil methanolic extracts [18]. Figure 4b shows the antioxidant activity index (AAI) of the four basil extracts, which is perfectly correlated with the IC_50_ value presented in Figure 4a.

A moderate antioxidant activity is represented by a value AAI between 0.5 and 1.0, a strong antioxidant activity is when the AAI value is between 1.0 and 2.0, and a very strong activity when AAI > 2.0 [23,32].

Of the electrochemical methods used for organic antioxidants analysis in foods or medicines, cyclic voltammetry is one of the most used because it is a fast and inexpensive method to detect antioxidants, such as phenols, carotenoids, or vitamins, in samples [33].

The cyclic voltammetry studies demonstrate that the electrochemical oxidation of the basil samples it is strongly related to the structure of the electroactive chemical compounds from the solvent extracts.

**Figure 9:**
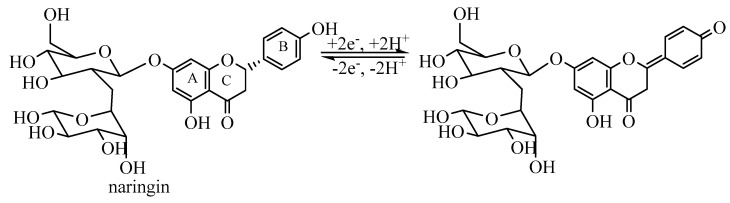
Naringin oxidative degradation.

The observed electrochemical process supports the good antioxidant activity of Romanian basil extracts which can be attributed to naringin. The naringin oxidative degradation exposed to electrochemical measurements is highlighted in Figure 9.

The literature data revealed that the conjugation of the ring A with the heterocyclic ring B and with the unsaturated ring C improves the antioxidant activity of this polyphenolic compounds. Also, the presence of the hydroxyl group in the 3 position of the heterocyclic ring increases the antioxidant activity of flavonoids. Lower antioxidant properties are exhibited by the flavonoid compounds in which no conjugation between the rings B and C is present. [34].

### 3.3. HPLC-DAD Separation, Identification and Quantification of Flavonoid Compounds

The chromatographic separation, using as reference compounds, naringin, naringenin, catechin (+), epicatechin (−), quercetin, hyperoside, rutin, tannic acid, caffeic acid, gallic acid, chlorogenic acid, p-coumaric acid, genistein, and daidzein, revealed the presence in the Romanian basil extracts of flavonoids, such as catechin (+), rutin, hyperoside, naringin, naringenin, and genistein (Figure 8). The flavonoid quantification revealed that the naringin is the principal compound of romanian hybrids basil extracts ranged from 9.30 ± 0.15 to 26.18 ± 0.23 mg/kg of dry plant. The best quantities of naringin were recorded for both methanolic extract of basil hybrids, the concentration being three times higher in *O. basilicum* variety “Aromat de Buzau”.

The identification of flavonoids argues for the antioxidant activity of basil alcoholic extracts and a proposed mechanism for this could include the following steps:

1. Dissociation of the antioxidant flavonoid ArOH:

(1) $$\begin{array}{ccccc} \mathrm{ArOH} & \to & {\mathrm{ArO}}^{-} & + & H^{+}\\ \mathrm{antioxidant} & \; & \mathrm{aroxyl}\;\mathrm{anion} & \; & \; \end{array}$$

2. The electron transfer from the antioxidant anion to the scavenged radical with the formation of the radical anion (R^−^):

(2) $$\begin{array}{ccccccc} {\mathrm{ArO}}^{-} & + & R^{\cdot } & \to & {\;\mathrm{ArO}}^{\cdot } & + & R^{-}\\ \mathrm{antioxidant}\;\mathrm{anion} & \; & \mathrm{free}\;\mathrm{radical} & \; & \; & \; & \mathrm{radical}\;\mathrm{anion} \end{array}$$

3. Radical anion protonation:

(3) $$\begin{array}{ccccc} R^{-} & + & H^{+} & \to & \mathrm{RH}\\ \mathrm{radical}\;\mathrm{anion} & \; & \; & \; & \; \end{array}$$

In the above-mentioned mechanisms, the hydroxyl group of flavonoids stabilize the ROS (reactive oxygen species), and the ionization degree of a polyphenolic antioxidant (ArOH) depends on characteristic properties of the solvent and their relative ability to solvate and stabilize antioxidant anions. The diversity of these polyphenolic secondary metabolites at the level of each species gives it a different antioxidant activity.

Recently, many studies suggest that an essential role in the defense against oxidative stress, at cellular level, plays the Nrf2, nuclear factor erythroid 2-related factor 2, it activates under various stress conditions, such as exposure to reactive oxygen species or electrophilic stress. For example, genistein, naringenin, and epigallocatechin gallate can induce Nrf2 expression in human breast carcinoma, Caco-2, or endothelial cells and protect them from oxidant injury [3,4,17,35,36] and it exhibits anti-oxidant, anti-mutagenic, and anti-carcinogenic effects [37,38].

**Figure 10:**
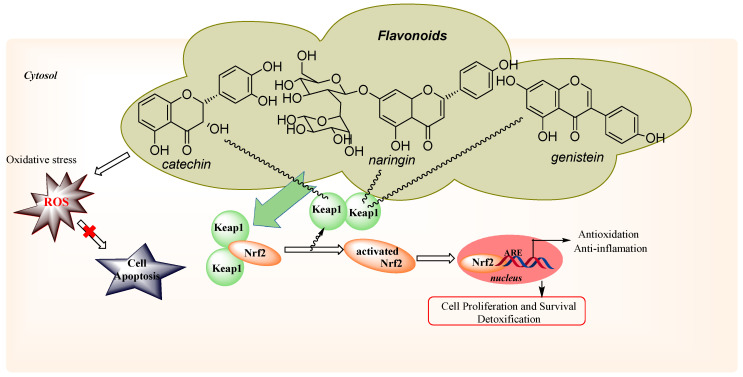
Schematic model representation of flavonoids mechanism involved in antioxidative defense.

At the cellular level, in the cytoplasm, normally, Keap1 (Kelch-like ECH-associated protein 1) is retained and bound to Nrf2. Under conditions of oxidative stress, the Nrf2-Keap1 complex is interrupted by inductors. Flavonoids act as inductors and are oxidized to its quinone derivatives. After, the corresponding quinone flavonoids form reacts in a Michael addition with Keap1. Nrf2 is released from Keap1, undergoes rapid translocation into the nucleus when bound to antioxidant response elements (AREs) and expresses phase II inducing cell detoxification and antioxidant proteins, such as hemoxygenase-1 (HO-1) and glutamate-L-cysteine ligase (GCLC) [35] (Figure 10).

## 4. Materials and Methods

### 4.1. Romanian Basil Hybrid Plant Materials

Two different selections of Romanian basil varieties named “Aromat de Buzau” (*O. basilicum*) and “Macedon” (*O. citriodorum*) were grown using organic production methods at the Vegetable Research and Development Station, Buzau, Romania. The mature plants used for the research investigation were harvested in early in August 2019.

### 4.2. Sample Preparation

The separation of chemical compounds, based on polarity, from plant tissue involves the use of selective solvents depending on the desired compounds [39]. The entire plants were dried at ambient temperature until the aerial parts became dried for grinding. After 3 weeks of drying, the basil plant materials (flower, seeds, and leaves) were ground using a mechanical blender into a fine powder. Methanol and ethanol were used as solvents for extraction of phytochemicals to obtain the maximum yield by using a reflux condenser. Subsequently, 10 g of basil powder and 100 mL of alcohol were added in a round bottom flask, and the samples were ultrasonicated for two hours. The resulted alcoholic extracts were filtered and concentrated in vacuum using a rotary evaporator to obtain the dry extract. The crude obtained extracts were stored at −18 °C until further use. The extraction was performed in triplicate.

### 4.3. Phytochemical characterization of the Romanian Varieties of Basil

#### 4.3.1. Phenolic Acids content

The total phenolic acids content was determined spectrophotometrically using Folin - Ciocalteu assay, according to the procedure described by Cudalbeanu, 2018 [23]. The Folin-Ciocalteu reagent reacts with phenolic compounds changing the color from yellow to blue. Briefly, 10 µL of basil sample was added to 25 µL of 1 N Folin–Ciocalteu reagent and allowed this mixture to stand for 5 min before adding 25 µL of 20% Na_2_CO_3_ aqueous solution. In the blank sample, the Folin–Ciocalteu reagent was replaced with alcohol. The absorbance value was measured versus the prepared blank at 760 nm, after 30 min at room temperature, using a Tecan Pro 200 apparatus, Tecan Trading AG multiwell plate reader (Männedorf, Switzerland). The total phenolic acids content was expressed as mg of gallic acid equivalents per gram sample (mg GAEq/g extract) through a calibration curve with gallic acid, and mg of tannic acid equivalents per gram sample (mg TAEq/g extract) through a calibration curve with tannic acid, respectively.

#### 4.3.2. Flavonoids Content

Briefly, 100 µL of 2% AlCl_3_ aqueous solution was added to 100 µL of sample. The absorbance value was read at 415 nm using the Tecan Pro 200 multiwell plate reader. For the blank sample, the basil sample was replaced with appropriate solvent. Quercetin and rutin were used as reference standards, and the results are given in mg quercetin equivalents per 1 g of sample, and mg rutin equivalents per 1 g of sample, respectively [23,40,41].

### 4.4. Antioxidant Potentials of the Romanian Varieties of Basil

#### 4.4.1. DPPH assay

Briefly, 100 µL of each basil sample was added to 100 µL DPPH (100 µg/mL). The samples were kept at room temperature in the dark for 20, 35, and 50 min. The UV-Vis spectra were recorded using Tecan Pro 200 multiwell plate reader (Männedorf, Switzerland). The absorbance values were recorded at 517 nm for each sample, compared with a blank sample (without extract). The DPPH inhibition percent was calculated using the formula: % inhibition=(Abs blank−Abs sample)/Abs blank×100. The IC_50_ values were graphically determined by plotting the inhibition percent against inhibitory concentration. The antioxidant activity index (AAI) was calculated using following formula: $\mathrm{AAI}=\mathrm{final}\;\mathrm{DPPH}\;\mathrm{concentration}/{\mathrm{IC}}_{50}\;\mathrm{value}\;$ [23,42].

#### 4.4.2. Electrochemical Evaluation of Antioxidant Capacity by Cyclic Voltammetry

The methanolic and ethanolic Romanian basil extracts were electrochemical evaluated using Biologic potentiostat/galvanostat SP-150 (Claix, France) at the room temperature. The electrochemical experiments were performed using an electrochemical cell with three electrodes: glass carbon electrode as working electrode, Ag/AgCl_sat_ (E = 0.194V/NHE) as reference electrode and Pt wire as counter electrode. The applied potential was E = ±1V vs. Ag/AgClsat, with rate potential at 100 mVs^−1^. The working electrode was polished with BASi^®^ polishing kit (alumina and diamond slurries) followed by washing with alcohol after each voltammetry experiment. Also, UV-Vis spectra were recorded for basil samples before and after cyclic voltammetry experiment, in the wavelength range from 200 to 500 nm, using the SPECORD 210 PLUS double-beam 500 spectrophotometer (Analytik Jena, Jena, Germany) [36].

### 4.5. HPLC-DAD Separation, Identification and Quantification of Flavonoid Compounds

The HPLC-DAD (high-Performance Liquid Chromatography with Diode-Array Detector) analysis was carried out using a High-Performance Liquid Chromatography Systems L-3000 (RIGOL TECHNOLOGIES, INC Beijing, China). In the chromatographic analysis, the Kinetex EVO C18 (150 4.6 mm, particle size of 5 µm) column was used with an injection volume of 10 µL. The solvents used were (A) 0.1% trifluoroacetic acid (TFA) in water and (B) 0.1% trifluoroacetic acid (TFA) in acetonitrile. The gradient elution was from 2% to 100% B at 30 °C for 60 min, and the elution flow was set at 1000 µL/min. Further, 300 nm analytical wavelength was used for detection in accordance with the literature [42].

### 4.6. Statistical Analysis

Three extractions were performed for each sample, and all assays were replicated three times for each sample. All the data experiments were expressed as mean ± standard deviation of the three independent assays. Data results were analyzed for statistical significance using the Microsoft Excel Program and Origin Pro 9.1.

## 5. Conclusions

The continuous research carried out in the field of natural compounds with antioxidants properties contributes to knowledge concerning the availability of natural and healthy compounds from herbal plants, which are particularly more attractive for commercial food producers and consumers than the synthetic antioxidants.

The results obtained in our study indicated that alcoholic extracts of the Romanian varieties of basil had moderate to high total phenolic acid content and good antioxidant activity against free radical DPPH in vitro assays. An evident correlation was found between the antioxidant capacity and the total phenolic content, and also a good correlation has been observed between the antioxidant capacities measured by means of the two techniques (DPPH assay and Cyclic Voltammetry).

The Romanian basil variety “Aromat de Buzau” showed the highest antioxidant activity and the highest contents of phenolic acid compounds. The major flavonoid found in both basil varieties was naringin, compound known for its pharmacologic activity such as hepatoprotective, hypocholesterolemic and antitumoral. In conclusion, our results partly justify the traditional uses of basil, with both Romanian *Ocimum* varieties being recommended to be a good dietary source of flavonoids. To validate the benefits of *Ocimum* extracts for human health and also for pharmaceutical industry, further work is necessary, and we have already started toxicity studies.

## Figures and Tables

**Table 1:** HPLC-DAD identification and quantification of flavonoids from basil extracts.

Compound (mg/kg)	TR * (min)	Aromat MeOH	Aromat EtOH	Macedon MeOH	Macedon EtOH
Catechin (+)	19.50	0.13 ± 0.01	0.23 ± 0.01	-	-
Rutin	29.78	0.78 ± 0.02	0.13 ± 0.01	-	0.31 ± 0.01
Hyperoside	29.98	-	-	-	0.20 ± 0.01
Naringin	31.50	26.18 ± 0.23	2.24 ± 0.09	9.30 ± 0.15	-
Naringenin	38.78	-	0.15 ± 0.01	0.17 ± 0.01	-
Genistein	39.10	0.50 ± 0.01	0.16 ± 0.01	-	0.17 ± 0.01

* Retention time (TR) error of mean for compounds was ±0.0001–0.2 min.-unidentified.
